# Immunodominant Antigens of *Leishmania chagasi* Associated with Protection against Human Visceral Leishmaniasis

**DOI:** 10.1371/journal.pntd.0001687

**Published:** 2012-06-19

**Authors:** Daniel R. Abánades, Leonardo V. Arruda, Elaine S. Arruda, José Roberto A. S. Pinto, Mario S. Palma, Dorlene Aquino, Arlene J. Caldas, Manuel Soto, Aldina Barral, Manoel Barral-Netto

**Affiliations:** 1 Centro de Pesquisas Gonçalo Moniz (CPqGM), Fundação Oswaldo Cruz (FIOCRUZ), Salvador, Bahia, Brazil; 2 Faculdade de Medicina da Bahia, Universidade Federal da Bahia, Salvador, Brazil; 3 Center of the Study of Social Insects, Institute of Biosciences of Rio Claro, Department of Biology, University of São Paulo State (UNESP), Rio Claro, São Paulo, Brazil; 4 Departamento de Enfermagem, Universidade Federal do Maranhão, São Luis, Maranhão, Brazil; 5 Centro de Biología Molecular Severo Ochoa (CSIC-UAM), Departamento de Biología Molecular, Universidad Autónoma de Madrid, Madrid, Spain; 6 Instituto Nacional de Ciência e Tecnologia de Investigação em Imunologia (iii-INCT), Salvador, Bahia, Brazil; Hospital Universitário, Brazil

## Abstract

**Background:**

Protection and recovery from visceral leishmaniasis (VL) have been associated with cell-mediated immune (CMI) responses, whereas no protective role has been attributed to humoral responses against specific parasitic antigens. In this report, we compared carefully selected groups of individuals with distinct responses to *Leishmania chagasi* to explore antigen-recognizing IgG present in resistant individuals.

**Methodology and Principal Findings:**

VL patients with negative delayed-type hypersensitivity (DTH) were classified into the susceptible group. Individuals who had recovered from VL and converted to a DTH+ response, as well as asymptomatic infected individuals (DTH+), were categorized into the resistant group. Sera from these groups were used to detect antigens from *L. chagasi* by conventional and 2D Western blot assays. Despite an overall reduction in the reactivity of several proteins after DTH conversion, a specific group of proteins (approximately 110–130 kDa) consistently reacted with sera from DTH converters. Other antigens that specifically reacted with sera from DTH+ individuals were isolated and tandem mass spectrometry followed by database query with the protein search engine MASCO were used to identify antigens. The serological properties of recombinant version of the selected antigens were tested by ELISA. Sera from asymptomatic infected people (DTH+) reacted more strongly with a mixture of selected recombinant antigens than with total soluble *Leishmania* antigen (SLA), with less cross-reactivity against Chagas disease patients' sera.

**Significance:**

Our results are the first evidence of *leishmania* proteins that are specifically recognized by sera from individuals who are putatively resistant to VL. In addition, these data highlight the possibility of using specific proteins in serological tests for the identification of asymptomatic infected individuals.

## Author Summary

One of the most striking features of infection by *Leishmania chagasi* is that infection leads to a spectrum of clinical outcomes ranging from asymptomatic infection to active disease. The existence of asymptomatic infected people has served as an incentive to believe that an effective vaccine is possible, but unfortunately no successful immunological characterization of such cases was obtained. Patients recovered from visceral leishmaniasis show a similar immunological profile to asymptomatic infected individuals and both exhibit a strong cell-mediated immune response against *Leishmania* antigens and are resistant to disease. Since the past decade several approaches were undertaken to try to shed light on the immunological profile associated with such “resistance” to infections, notwithstanding antigenic recognition profile associated to resistance to infection was not successfully explored. In the present manuscript we describe a specific IgG recognizing pattern associated with resistant individuals (asymptomatic infected people and recovery patients to visceral leishmaniasis). These data highlight the possibility of using specific proteins in serological tests for the identification of asymptomatic infected individuals.

## Introduction

Visceral Leishmaniasis (VL) is a potentially fatal disease caused by infection with *Leishmania chagasi* in the New World and *Leishmania donovani* or *Leishmania infantum* in the Old World [1]. Infection leads to a spectrum of clinical outcomes ranging from asymptomatic infection to active disease. The anti-*Leishmania* immune response during asymptomatic infection is characterized by a low serological and positive cellular response, which is demonstrated by a positive delayed-type hypersensitivity skin response (DTH+) [2]. Patients with active VL, on the other hand, present a strong positive serological and a negative cell-mediated immune (CMI) response with low IFN-γ production [3]. Treated patients that recover from illness (accounts for 90%) only exhibit a positive DTH response long after treatment [4]. Epidemiological studies in Brazil showed that a positive DTH response is a marker of protection against VL [5].

Serological diagnosis of *L. chagasi* during asymptomatic infection is complicated by low antibody titers and frequent cross-reactivity with other diseases [6]. In addition, serologic markers of recovery or resistance to infection have not been characterized. There is no effective and safe vaccine approved for human use against any form of visceral leishmaniasis despite the obvious need and considerable effort that has been made.

In the present report, we compared the reactivity against total protein extracts from *L. chagasi* of sera obtained from either DTH positive patients (asymptomatic or treated and recovered individuals) or symptomatic patients. A serological pattern associated with DTH positivity was observed in both asymptomatic individuals and in recovered patients. In addition, the recombinant version of select antigens appeared to be a valuable tool for the serological identification of asymptomatic patients.

## Materials and Methods

### Ethics statement

This study was approved by the Research Ethics Committee of the Federal University of Maranhão University Hospital, Brazil (localities where the field study was performed). All clinical investigations were conducted according to the Declaration of Helsinki. Written, informed consent was obtained from all participants or legal guardians.

### Parasites

*Leishmania chagasi* (MHOM/BR00/MER/STRAIN2) promastigotes were cultured in Schneider's medium supplemented with 10% inactive FBS, 2 mM L-glutamine, 100 U/mL penicillin, and 100 µL/mL streptomycin.

### Patients, sera and delayed-type hypersensitivity reaction

Sera were obtained at two distinct settings as described below: The patients were classified as VL (pre-treatment, samples obtained during active disease previous to treatment) and post-treatment (samples from treated and cured patients). All patients were from the Maranhão Federal University Hospital. Diagnosis was confirmed by identification of *Leishmania sp.* in Giemsa-stained smears of bone marrow aspirates (parasitological test). The study was conducted from August 2000 to July 2002 and information on the individuals has been previously reported [4], [7].

Sera were stored at −20°C without thawing. All patients received adequate treatment. DTH skin reactivity assays (Montenegro test) were performed with SLA prepared as described elsewhere [8].

### Sample preparation

Parasites in logarithmic growth phase were washed twice with PBS, lysed with Laemmli's Buffer [9], sonicated, heated at 95°C for 5 min and centrifuged for 15 min at 12,000×g at 4°C. Extracts from 10^6^ parasites were loaded by line into an acrylamide gel. For 2D electrophoresis, parasites were lysed at 4°C with 200 µL of Buffer A (0.5% Nonidet P40, 0.1 mM PMSF, 1 mM DTT, 10 mM Tris-HCl, pH 7.4) followed by addition of 200 µL of phenol and vortex. The samples were centrifuged at 6,000×g for 5 min, and the aqueous phase was discarded. The proteins were precipitated by adding 1 mL of 0.1 M ammonium acetate in absolute methanol and centrifuged at 6,000×g for 10 min at 4°C. The resultant pellet was washed with 80% acetone and dried. The proteins were solubilized for 3 h at 30°C in RP3 Buffer (7 M urea, 2 M thiourea, 4% CHAPS, 40 mM Tris-HCl pH 8.8, 0.5% ampholytes, pH 4–7), followed by 15 min of centrifugation at 12,000×g at 4°C. The amount of proteins in the supernatants was quantified using the Quick Start Bradford Protein Assay (BioRad. USA), and the proteins were then stocked at 20°C.

### 2D gel electrophoresis

Samples containing 250 µg *L. chagasi* protein extract in 200 µL RP3 Buffer supplemented with DTT (50 mM) were applied by rehydration to 11 cm IPG strips (pH 4–7). Isoelectric focusing (IEF) was performed using a Multiphor II electrophoresis unit (GE Healthcare, UK) at 3,500 V for 15,000 Vh. Subsequently, the IPG strips were reduced (130 mM DTT) and alkylated (135 mM iodoacetamide) for 15 min in equilibration buffer (0.375 M Tris-HCl, pH 8.8, 6 M urea, 20% vol/vol glycerol, 2% wt/vol SDS). The second dimension was run on home-casted SDS-PAGE gels (10% or 8% wt/vol polyacrylamide) at 50 V for 30 min and then at 160 V until the dye front reached the bottom of the gel. With the separation in the second dimension, the proteins were visualized by staining with PlusOne™ Silver Staining Kit or Colloidal Coomassie staining (GE Healthcare, UK).

### Western blot

The electrophoresed proteins were transferred to nitrocellulose membranes (GE Healthcare, UK) and were stained with Ponceau S. Membranes were blocked with 5% non-fat dried milk powder in wash solution (PBS and 0.05% Tween 20). The membranes were probed with sera (1∶1000 or 1∶500), and an anti-human-IgG Phosphatase Alkaline (PA) immunoconjugate (Sigma-Aldrich. Germany) was used as a secondary antibody (1∶2000). To measure the recognition of sera by IgG subclasses after incubation, mouse anti-human-IgG1, IgG2, IgG3 and IgG4 were employed. After three washes with wash solution, an anti-mouse-IgG PA immunoconjugate was used (1∶2000). Specific IgG-PA binding was measured with Western Blue® Stabilized Substrate for Alkaline Phosphatase (Promega, USA).

### Matching antigens and protein spots

To match antigen spots in Western blots with the corresponding protein spot in the Coomassie gels, the blot coordinates were defined after the Ponceau S staining pattern of the blot filter was aligned with the spot pattern of the Coomassie gel. Only perfect overlap (position and form) between blot-spot and Ponceau S staining-spot was accepted (mapped spot). Spots in Western blots without overlap with Ponceau S or Coomassie spot were defined as unmapped spots.

### In-gel digestion

The protocol that was used for in-gel digestion was based on that in a previous publication [10]. Briefly, gel pieces were distained twice for 30 min at 25°C with 25 mM ammonium bicarbonate/50% (w/v) acetonitrile, dehydrated in acetonitrile, dried, and treated with trypsin (20 µg/mL, Promega, USA) in 25 mM ammonium bicarbonate pH 7.9 at 37°C for 16 h. Digests were extracted from gel pieces with 50% (v/v) acetonitrile/water and 0.1% (v/v) formic acid and subsequently combined and vacuum-dried. The concentrated digests were mixed with 0.5 µL of α-cyano-4-hydroxycinnamic acid matrix (10 mg/mL) in 50% (v/v) acetonitrile/0.1% (v/v) trifluoroacetic acid and were spotted onto a MALDI target plate.

### MALDI-ToF/ToF mass spectrometry data

Mass spectrometric analysis was performed using MALDI ToF/ToF-MS/MS (matrix-assisted laser desorption ionization time of flight/time of flight-mass spectrometry) on a Shimadzu instrument (model Axima Performance). MS data were acquired in the m/z range of 700 to 4,000, with an accelerating voltage of 20 kV, delayed extraction, a peak density of maximum 50 peaks per 200 Da, a minimal S/N ratio of 10 and a maximum peak at 60. MS/MS data were acquired in the mass range of 60 Da to each precursor's mass, with a minimum S/N ratio of 10, a maximum number of peaks set at 65 and a peak density maximum of 50 peaks per 200 Da.

### Protein identification

LaunchPad 2.8.4 (Shimadzu Biotech) was used to submit the combined MS and MS/MS data to the MASCOT protein search engine version 2.2 using the National Center for Biotechnology Information (NCBI) protein database. The search parameters were as follows: no restrictions on protein molecular weight; one tryptic missed cleavage allowed; peptide mass tolerance in the searches was 0.2 Da for MS spectra and 0.8 Da for MS/MS spectra. Carbamide-methylation due to treatment of sulfhydryl with iodoacetamide and oxidation of methionine and tryptophan were specified in MASCOT as fixed and variable modifications, respectively.

### Cloning and recombinant protein purification

For expression of antigens identified by MALDI ToF/ToF-MS/MS, coding regions were amplified by polymerase chain reaction (PCR) and were subcloned into the pQE30 expression vector (Qiagen, Germany). The following primers were employed for amplification: Enolase, forward: 5′-CGGGATCCATGCCGATCCAAAAGGTTTAC-3′ and reverse: 5′-CCAAGCTTTTACGCCCAGCCGGGGTAG-3′; S-adenosylmethionine synthetase, forward: CGGGATCCATGTCTGTCCACAGCATCCTC, and reverse: 5′-CCCAAGCTTTTACTCGACCATCTTCTTGG-3′; Alpha tubulin, forward: 5′-CGGGATCCATGCACACAGACACGCACGC-3′, reverse: 5′-GGGGTACCCCTTCGCTTCACTATTTTTG -3′; Heat shock protein 70, forward: 5′-CGGGATCCATGTCGTCTACCAACGCCATC-3′, reverse: 5′-CCCAAGCTTTTAGTCAACGTCTTCGGCG-3′; Heat shock 70, mitochondrial precursor, forward: 5′-CGGGATCCATGTTCGCTCGTCGTGTG-3′, reverse: 5′-GGGGTACCTCAACTATTACCTGAGTAGG-3′ and heat shock protein 83-1, forward: 5′-CGGGATCCATGACGGAGACGTTCGCGTT-3′, reverse: 5′-CCCAAGCTTTCAGTCCACCTGCTCCATGC-3′. Underlined sequences in primers indicate restriction sites for cloning.

Recombinant antigens were over-expressed in *E. coli* cultures, transformed with serial pQE30s by the addition of 2 mM isopropyl β-D-1-thiogalactopyranoside (IPTG), followed by 3 h at 37°C incubation. Non-native bacterial lysates were subjected to Ni-nitrilotriacetic acid agarose columns chromatography (Qiagen, Germany). Purification was performed according to the manufacturer's instructions.

### ELISA

Soluble *Leishmania* antigen (SLA) from *L. chagasi* and recombinant purified proteins were diluted in PBS buffer to 1 µg/100 µL, and then 100 µL of each sample were placed into wells of 96-well microtiter plates (Probind; Falcon, Becton Dickinson, USA) and were incubated overnight at 4°C. Wash solution (PBS 1× with 0.5% Tween 20) was used three times for 10 min at room temperature. To block wells, a blocking solution (PBS plus 0.5% Tween 20 and 5% non-fat milk) was used for 1 h at room temperature. Serum samples diluted at 1∶100 in blocking solution were added at 100 µL/well, and plates were then incubated for 2 h at room temperature. A new round of washes was performed as indicated previously, followed by an incubation of 1 h at room temperature with a 1∶2000 dilution of alkaline phosphatase-conjugated anti-human IgG antibody (Sigma-Aldrich, Germany). Antibody excess was removed by four rounds of 10 min washes using wash solution. The plates were developed using a chromogenic solution of p-nitrophenylphosphate in sodium carbonate buffer (pH 9.6) with 1 mg/mL MgCl_2_. The absorbance was recorded at 405 nm.

## Results

### IgG reaction with total *L. chagasi* protein in patients during DTH conversion

**Figure 1:**
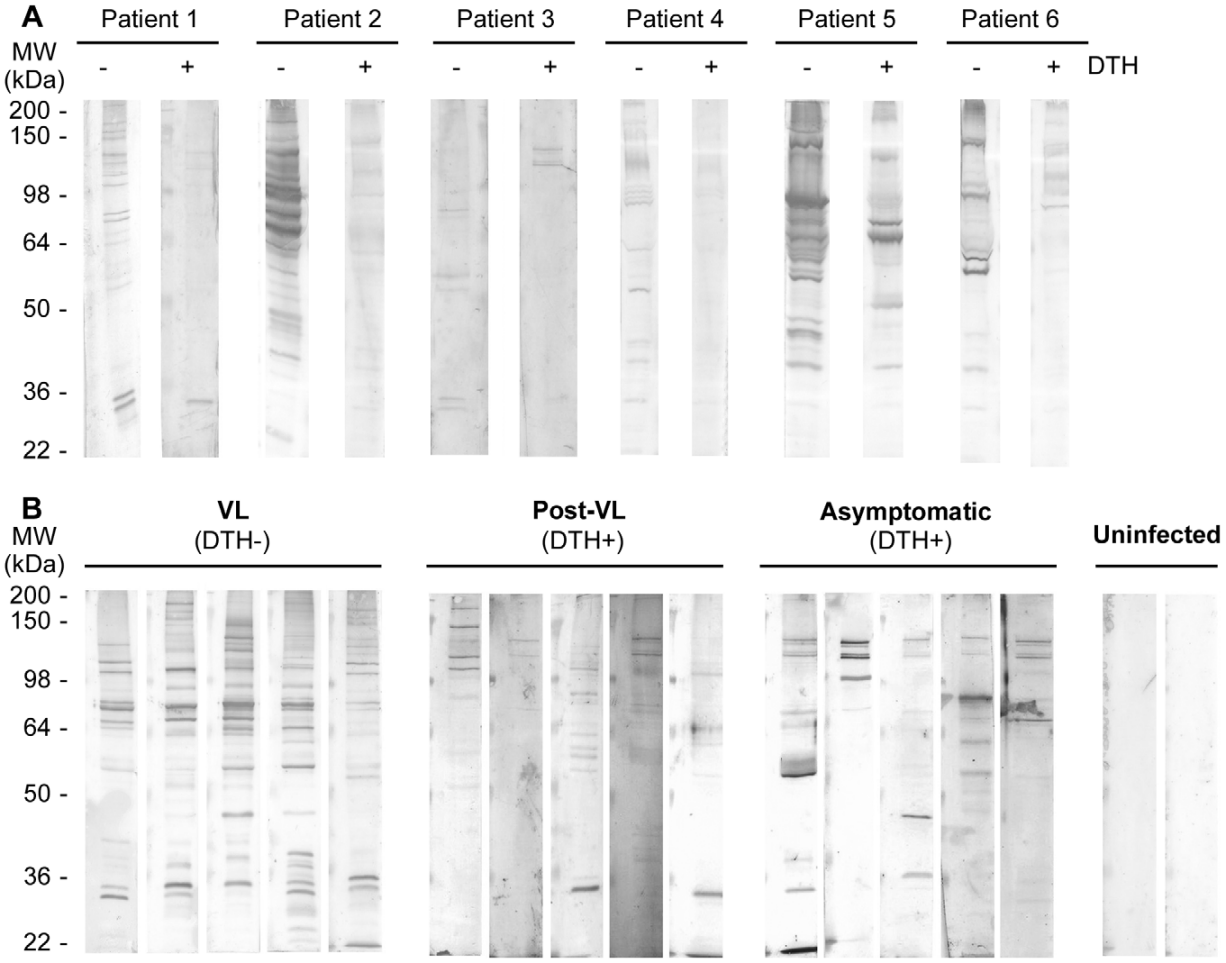
IgG reaction pattern associated to DTH conversion. Total protein extract from *L. chagasi* was SDS-PAGE resolved (10% polyacrilamide) and electrotransferred to nitrocellulose filter. In all blots, sera were used at 1∶1000, and secondary anti-human IgG was used at 1∶2000. A) Sera from six VL patients before and after DTH+ conversion. B) Western blot results using sera from active VL and recovered VL patients, asymptomatic infected and non-infected individuals. Molecular weight markers are shown on the left panel.

After treatment, VL patients develop anti-*Leishmania* CMI, as evidenced by positive Montenegro reaction followed by decreases in titers of IgG against *Leishmania* total protein [2]. However, even a year after treatment and curing disease, anti-*Leishmania* antibodies are still present in the sera [4]. To understand if this reduction in IgG recognition is associated with changes in antigen specificity, we screened sera from patients before and after DTH conversion for reactivity with *L. chagasi* total protein by Western blot. A decrease in the number of proteins with reactivity was observed in sera from VL patients after their recovery, and new reactivity patterns were observed after DTH conversion (Figure 1A). Next, we compared the reactivity pattern of sera from symptomatic VL patients (DTH−), recovered patients (DTH+), asymptomatic donors (DTH+) and uninfected volunteers from the same endemic area (Figure 1B). Sera from post-treatment and asymptomatic patients both of which were DTH+, showed weak or no reactivity to a majority of the bands. A high mass proteins group (approximately 110–130 kDa) was detected in all naturally resistant patient samples and in 60% of post-treatment DTH+ sera tested (Figure 1; Supplementary Figure S1; Baseline characteristics of the study population used in Table S1). To determine if any IgG subclass was involved in the differential reactivity of sera between groups, IgG1, IgG2, IgG3 and IgG4 binding was tested. Only IgG1-antibody showed significant reactivity that was similar to that of the total IgG pattern in all cases (Table S1).

### Characterization of 2D IgG recognizing by sera of VL and DTH+ patients

**Figure 2:**
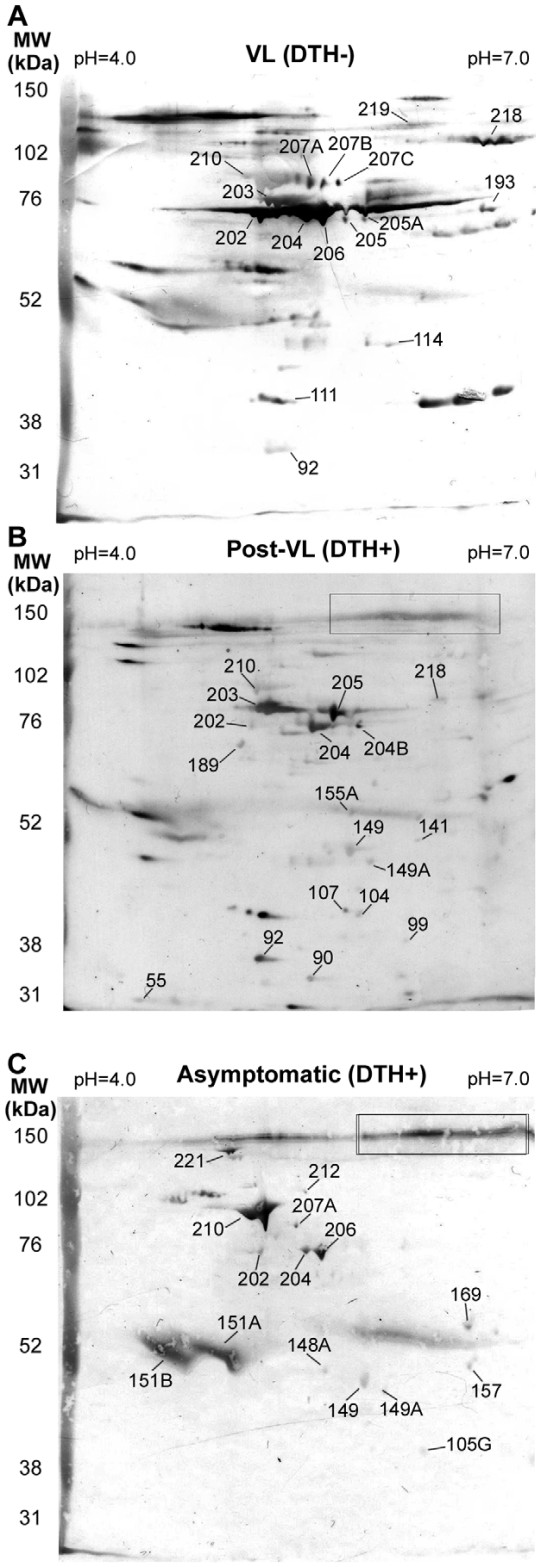
2D Western blot using sera from VL (DTH−), post-VL (DTH+) and naturally resistant individuals (DTH+). Total protein extract from *L. chagasi* were added to 11 cm strips (pH 4–7), followed by SDS-PAGE (8% polyacrylamide). After Ponceau staining, the filters were incubated with a mixture of sera at 1∶1000 from VL (A) and 1∶500 from post-VL (B) and naturally resistant donors (C). Only mapped spots are indicated. Numbers corresponding with spots from 2D maps (supplementary Figure S2). A specific DTH+ signal (none mapped) is shown in the designated box. MW, Molecular weight markers.

To characterize the reactivity patterns associated with protection against VL, 200 µg of total *L. chagasi* protein was 2D electrophoresed, electrotransferred to nitrocellulose membranes and tested against serum samples. After the second dimension (using 10% or 8% SDS-PAGE gel), approximately 250 spots were obtained that had high resolution and reproducibility (Figure S2). Membranes were tested using a pool composed of the five more reactive sera from each group, and IgG interactions were detected. Strikingly, we observed significant differences between groups in the 2D Western blot (Figure 2). Results were summarized in Table 1. In VL, post-treatment VL and asymptomatic patients, 58, 62 and 33 spots were detected respectively (Table 1). It is remarkable that the ratio between mapped spots versus total spots reactions were higher when we used sera from asymptomatic (14/33) than those found we used sera from VL (15/58) or Post-VL (17/62) groups. Each group showed specific mapped spots, whereas two mapped spots (202 and 204) were recognized by all groups. In asymptomatic individuals, spots mapped were not detected in the 110–130 kDa range, but a specific non-mapped signal was detected (delineated in a quadrant in Figure 2). This signal was also recognized by sera from a post-treatment VL sample; however it was not observed when we used VL patient sera.

**Table 1:** Summary of 2D Western blot results.

	VL	Post-VL	VL-Resistant
**Total Spot Reactions**	58	62	33
**Specific Mapped Spots**	219	189	210
	207B	155A	212
	205A	141	151B
	193	107	151A
	114	104	148A
	111	99	169
		90	157
		55	105G
**Non-Specific Mapped Spots**	202	202	202
	204	204	204
	207A		207A
	206		206
	218	218	
	210*	210*	
	203A/B	203A/B	
	205	205	
	92	92	
		149	149
		149A	149A
**Total Mapped Spots**	15	18	14
**Total Unmapped Spots**	43	44	19

Reproducible spots reacting with each group are indicated. Mapped and non-mapped spots are defined in Materials and Methods. Asterisks indicate low recognition.

MALDI ToF/ToF-MS/MS analysis of the 24 identified spots (of which 6 spots were specifically recognized by VL patient sera, 8 spots by post-treatment VL sera, 8 spots by asymptomatic patient sera and 2 spots recognized by all groups) resulted in the identification of 14 (58.3%) proteins. These results, combined with MASCOT protein identification, are summarized in Table 2.

**Table 2:** Summary of MALDI ToF/ToF-MS/MS results.

	Spot	Uniprot (Acession Number)	Name Function/activity	*Mascot Score	Sequence Coverage %	MW (kDa)	pI	MS/MS peptide (Ion Score)	References
**VL**	205A	A415TO	HSP 70-like mt	109	25	72	5.7	K.EINDVVLVGGMTR.I (20)	-
	207B	Q66V59	Paraflagellar rod protein 1	61	18	69.6	5.3	-	-
**Post-VL**	55	A4IDB2	Translation elongation factor 1-beta	76	17	23,2	4.6	R.LNAQPFVSGFSPSSEDAR.I (48)	[15]
								R.IFNEMFGSNVNVIQWVAR.M (28)	
	141A	Q25225	Eukaryotic initiation factor 4A	94	3	45.3	5.8	R.GIYSYGFEKPSSIQQR.A (94)	[12], [26]–[28]
	149	A4HW62	Enolase	54	28	46	5.3	-	[13], [16], [28], [29]
	149A	A4I641	S-adenosylmethionine synthetase	58	28	43.5	5.5	R.RPIYYETSR.F (27)	[14]
								R.FGHFGR.K (43)	
	189	A4IAG8	Adaptor gamma-1 chain	23	1	90.7	5.3	R.RALDLTVTLITVNNVR.L (23)	-
**VL Resistant**	148A	Q8I8E1	Disulfate isomerase PDI	60	2	52.8	5.2	K.FPAFVVDFER.R (24)	[20], [29]
	151B	P21148	Tubulin, beta chain	124	7	50.6	4.7	R.FPGQLNSDLR.N (48)	[13], [18], [27], [30]
								R.LHFFMMGFAPLTSR.G (5)	
								R.YLTASALFR.G (71)	
	151A	Q8ITR7	Tubulin, alpha chain	50	3	41.0	6.2	R.TIEFVDWCPTGFK.C (56)	[13], [19]–[21], [29]
	157	A4I3X4	Vacuolar ATP synthase subunit B	128	8	55.6	5.8	R.IFNGSGIPIDNGPPVLPEQFR.N (92)	[22]
								K.IPLFSGAGLPHNEIAAQIVR.Q (36)	
	210	A4I8Q0	Hsp 83-1	52	14	79.4	5.1	K.HFSVEGQLEFR.S (22)	[17], [31], [32]
**All**	202	A4I253	HSP 70-related protein	80	20	70.8	5.1	-	
	204	Multiplex	70 kDa heat shock protein	246	7	56.7	6.4	R.LVTFFTEEFK.R (50)	[22], [24], [33], [34]
								R.LVTFFTEEFKR.K (72)	
								R.LVTFFTEEFKR.K (44)	
								R.ARFEELCGDLFR.S (52)	
								K.SQIFSTYADNQPGVHIQVFEGER.A (72)	

**Note.** References are related to the significance of these antigens with regard to drug resistance, host parasite survival or immunological properties.

*: Mascot Score, protein overall scores greater than 20 are significant (P<0,05). MW, molecular weight; Ip, isoelectric point.

We identified the following two proteins that specifically reacted with sera from VL patients (DTH−): Mitochondrial 70 kDa heat shock protein (MPT70, spot 205A) and paraflagellar rod protein 1 (PFR1, spot 207B). Additionally, eleven proteins specifically reacting with sera from DTH+ patients were identified. Five proteins reacting with sera from post-VL treatment patients (translation elongation factor 1-beta [LieEF1B, spot 55], eukaryotic initiation factor 4A [LieIF4A, spot 141A], enolase [spot 149], S-adenosylmethionine synthetase [MAT2, spot 149A] and adaptor gamma-1 chain [spot 189]) and 6 proteins with specific reactivity with sera from asymptomatic infected patients (disulfate isomerase [PDI, spot 148A], alpha- and beta-tubulin [spots 151A and 151B, respectively], vacuolar ATP synthase subunit B [spot 157] and 83 kDa heat shock protein [HSP83, spot 210]) were identified. Finally, the two proteins recognized by all groups (spots 202 and 204) were both identified as 70 kDa heat shock protein (HSP70). The putative functions and immunological properties of the identified proteins were retrieved from published literature and also from the *L. infantum* Genome Project database (www.genedb.org).

### Characterizing the humoral response against recombinant proteins from *L. chagasi* identified by proteomic approaches

**Figure 3:**
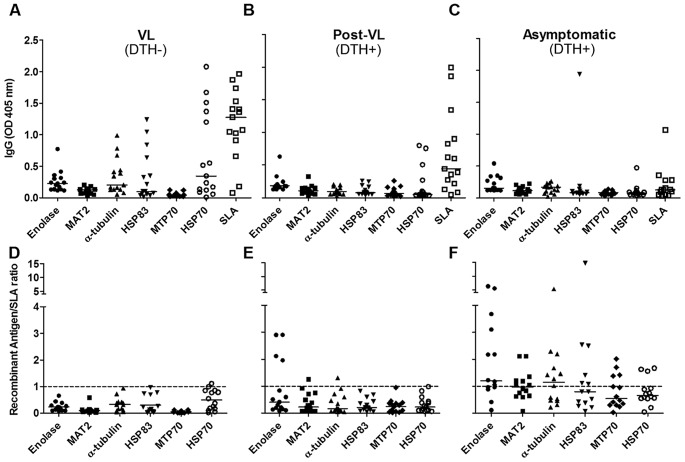
ELISA with individual recombinant proteins. SLA and obtained recombinants proteins (10 µg/mL) were tested for reactivity with sera (diluted 1∶100) from those groups shown above the panels. Absolute OD obtained (A–C) and the ratio between antigen-OD versus SLA-OD for each serum sample (D–F)) are shown. The dotted line shows the OD ratio at 1. Medians are shown as horizontal lines.

**Figure 4:**
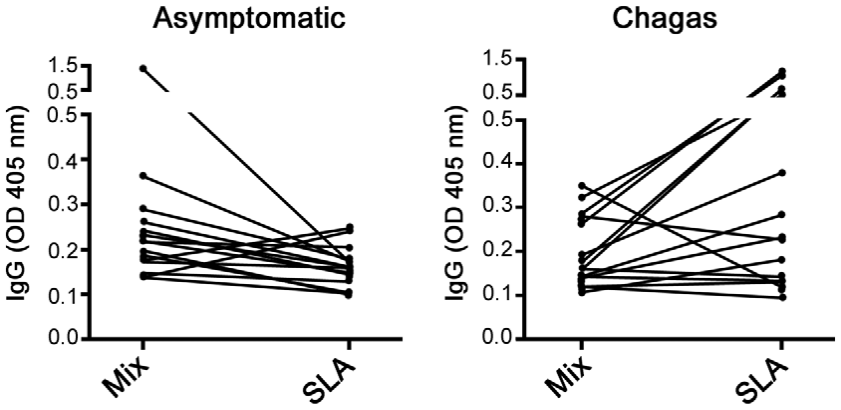
Mixture of DTH+ antigens in serological diagnosis of asymptomatic infected individuals. ELISA results were obtained using 10 µg/mL SLA or DTH+ recombinant antigen mixture (MIX, contained 2.5 µg/mL of each of the following proteins: enolase, MAT2, alpha tubulin and HSP83). Sera from asymptomatic *L. chagasi* infected patients, Chagas-infected patients were used (1∶100).

To analyze whether the identified proteins were indeed reactive with sera from the respective groups and could be used in a serological test for potentially asymptomatic *L. chagasi* infected individuals, we measured reactivity of the following recombinant proteins (Figure S3 for supporting information) by ELISA: alpha-tubulin, enolase, MAT2, and HSP83, which were reactive with DTH+ individuals sera; MPT70, which reacted with VL patients' sera and HSP70, which reacted with sera from both groups (Figure 3). Recombinants antigens reacted with some of the serum samples from VL (DTH−), post-treatment (DTH+) and asymptomatic patients (DTH+) (Figures 3 A–C). However, when we normalized the titers of IgG reactive with antigens from SLA, we observed that asymptomatic patients presented higher specific reactivity with DTH+ antigens (Figure 3D–F). We then used a combination of recombinants proteins composed of enolase, MAT2, alpha-tubulin and HSP83 (MIX) to test the sensitivity in the sera from asymptomatic patients and their specificity against sera from patients with Chagas disease, which presents high cross-reactivity with *Leishmania* antigens [11]. We verified that asymptomatic individuals had a higher reaction with MIX than SLA, and sera from patients with Chagas disease showed lower cross-reactivity (Figure 4).

## Discussion

The decrease in anti-*Leishmania* IgG titers during conversion to DTH+ in effectively treated VL patients has been previously associated with the development of cell-mediated immune responses [4]. Herein, we show that decreased antibody levels in VL-related states and positive DTH reactions are associated with a reduction in the reactivity against most of the parasite proteins and, more relevant, the recognition of previously unrecognized proteins. These observations indicate that important changes in the humoral response occurs during DTH conversion and reveals a reactivity pattern associated with recovery and natural resistance to VL that is characterized by high-mass group proteins (approximately 110–130 kDa). These high-mass proteins did not react with sera from VL patients, indicating that some specific immunodominant antigens are specifically associated with DTH conversion and putatively with protection against VL. Unfortunately, high-mass group proteins were not 2D-resolved, and their identification was not possible; however, 2D-recognizing map analyses of sera from VL patients and DTH+ individuals (post-VL or naturally resistant) showed other immunodominant antigens whose recognition was DTH status-dependent. A lower number of reactive spots were observed in sera from DTH+ individuals living in the endemic area who are presumably naturally resistant. It is noteworthy that sera from post-VL patients (DTH+) reacted with a larger number of proteins, which suggests that post-VL patients likely share antigenic recognition patterns with both VL patients and naturally resistant individuals which reinforces the idea that some antigens are specifically associated with positive DTH response and therefore protection against VL.

Proteomic analysis revealed the identification of 14 antigens, including proteins not previously reported as antigenic, such as adaptor gamma-1 chain, MPT70 and PFR1. Among the identified antigens associated with the post-VL subset, four of them (LieIF4A, LieEF1B, enolase and MAT2) have been reported as highly expressed in drug-resistant strains [12]–[14] and have been implicated in the survival of the parasite inside the host [15], [16]. For the six identified antigens that reacted with sera from the VL naturally resistant people (PDI, alpha- and beta-tubulin, vacuolar ATP synthase subunit B and HSP83), drug resistance implications and host parasite survival properties have been previously described too [13], [17]–[21]. The stress state imposed on the parasite in a DTH+ environment (resulting either from drug treatment or natural resistance) can induce the overexpression of these proteins, and this fact may explain their immunodominant antigenicity in these individuals. It is notable that LieIF4A protein was described as antigen reactive with sera from VL patients infected by *L. donovani* from India [25] and we found a clear reactivity against this protein using sera from post-VL patients. It could be interpreted as yet another indication that IgG pattern recognition in post-VL patients preserved reactivity against some antigens of active illness. In addition, it was already observed that a Th1 activation response, associated with healing, was induced in cutaneous leishmaniasis patients' PBMC by recombinant LieIF4A [26]. We consider the hypothesis that some correlation may exist between the antigenicity of DTH+ recognized proteins and its capacity to induce anti-*Leishmania* cellular responses. On the other side, it is possible that the highly immunogenic proteins, such as HSP70 [22]–[24], are implicated in its recognition by all groups.

To determine if the immunodominant antigens identified could serve as serological markers, some of them were expressed as recombinant proteins, purified and then employed as antigen in ELISA (Figure 3). When we normalized to SLA, enolase exhibited a remarkable increase in reactivity against sera from post-VL group respect to other antigens. Moreover, an increased enolase recognition was observed using sera from asymptomatic people, strengthening the idea that antigens recognized by post-VL sera could also be reactive in VL patients and resistant people sera. In the other hand, alpha tubulin and HSP83, showed a remark recognition increase from asymptomatic donors' sera. Meanwhile, MTP70 that was identified as a VL-specific protein did not react with any groups. There are four not identical copies of MPT70 in *L. infantum* genome (www.genedb.org), and only one of them was cloned to obtain MPT70 recombinant protein. Future studies with the other 3 variants will shed light about the real serological properties of these proteins. Finally, and as expected, HSP70 reacted with several serum samples from all groups.

Lastly, we proved that a mixture of DTH+ antigens (enolase, MAT2, alpha-tubulin and HSP83) had higher reactivity with sera from asymptomatic individuals than SLA and lower cross-reactivity with sera from patients with Chagas disease. Our results are the first description of several antigens that are immunodominant in DTH+ individuals (post-VL and naturally resistant people). Future studies using these antigens may help to identify potent serological tools that could be useful for determining patient disease status as well as new anti-*Leishmania* vaccine candidates.

## Supporting Information

Figure S1 Click here for additional data file. **Characterization of IgG reaction pattern associated to DTH conversion.** Total protein extract from *L. chagasi* was SDS-PAGE resolved (10% polyacrilamide) and electrotransferred to nitrocellulose filter. In all blots, sera were used at 1∶1000 (1∶100 in B - line 5) and secondary anti-human IgG (or subclasses IgG1, IgG2, IgG3 and IgG4 in E and F) was used at 1∶2000. A) Sera from VL patients. B) Sera from uninfected people. C) Sera from post-VL patients (DTH+). D) Sera from asymptomatic infected people. E) VL sera mix (VL0678, VL0669, VL0660, VL0671, VL0666). F) Asymptomatic sera mix (576, 421, 689, 011, 417). Under panels are show the serum code. Asterisk over panel indicate filters with DTH+ patter (110–130 KDa proteins). MW, Molecular weight markers. Baseline characteristics of the study population are shown in supplementary Table S1.
(TIF)

Figure S2 Click here for additional data file. **2D proteomic map of *L. chagasi*.** Approximately 200 µg of total protein from *L. chagasi* was 2D resolved on 11 cm strips with a pH range of 4–7 (first dimension) and SDS-PAGE. The numbers of mapped spots are indicated. A) Gel after electrotransfer to a nitrocellulose filter and Ponceau Red staining (8% polyacrylamide in second dimension). B) Gel with 10% polyacrylamide and Colloidal Coomassie staining. C) Gel with 10% polyacrylamide and Silver staining.
(TIF)

Figure S3 Click here for additional data file. **Purified recombinant antigens of *L. chagasi* identified by proteomic approaches.** Identified *E. coli* (M15) overexpressing recombinant antigens (fused to a 6xHis tag) were used for Ni-affinity chromatography. Obtained purified proteins resolved in 10% polyacrylamide gel stained with Comassie are shown. MW, Molecular weight marker.
(TIF)

Table S1 Click here for additional data file. **Baseline characteristics of the sera donors used in this study.** Age, sex, serum code, OD value obtained from Elisa test, and DTH status for each of the groups are show.
(DOCX)
