# Supplementary figures and images for: Immunodominant Antigens of Leishmania chagasi Associated with Protection against Human Visceral Leishmaniasis
Source: PLoS Negl Trop Dis. 2012 Jun 19;6(6):e1687. doi: 10.1371/journal.pntd.0001687 (PMC3378602; doi:10.1371/journal.pntd.0001687)

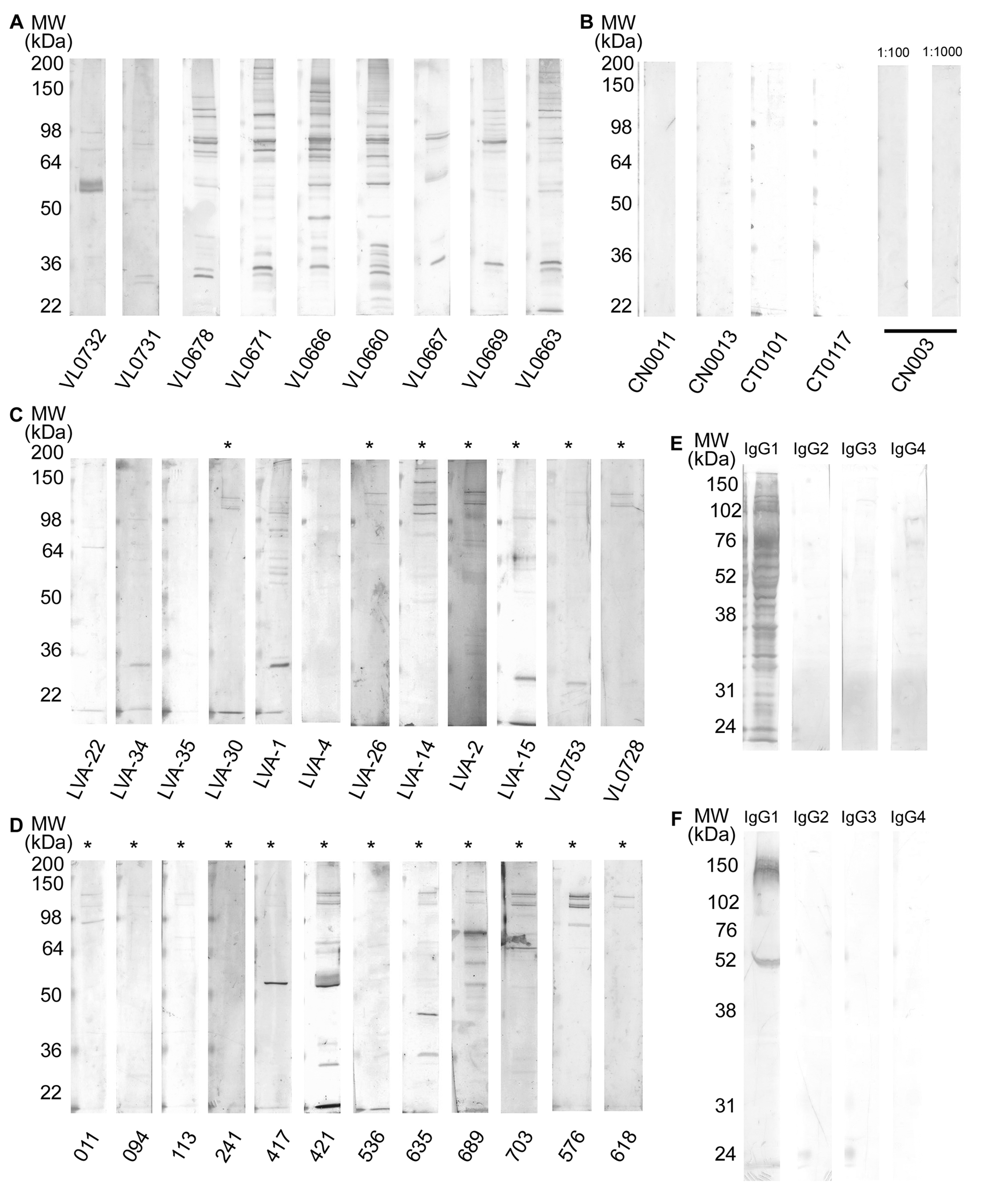

Supplement: Figure S1 [file pntd.0001687.s001.tif]

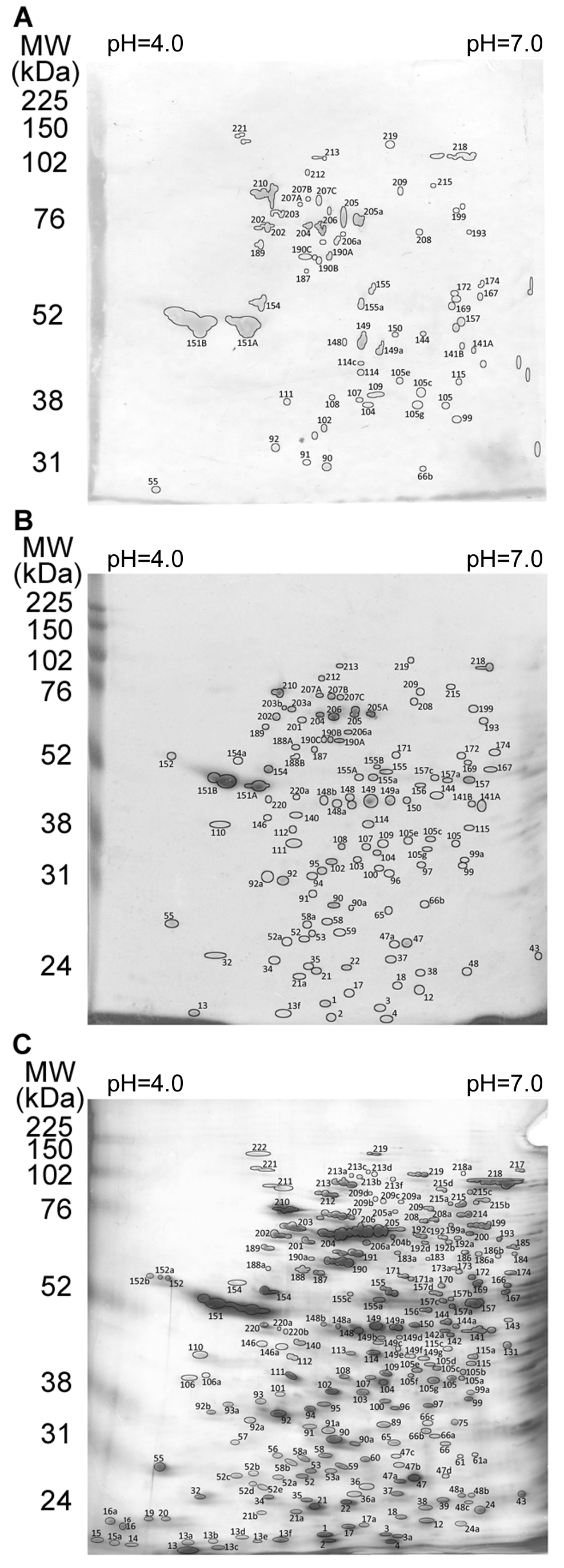

Supplement: Figure S2 [file pntd.0001687.s002.tif]

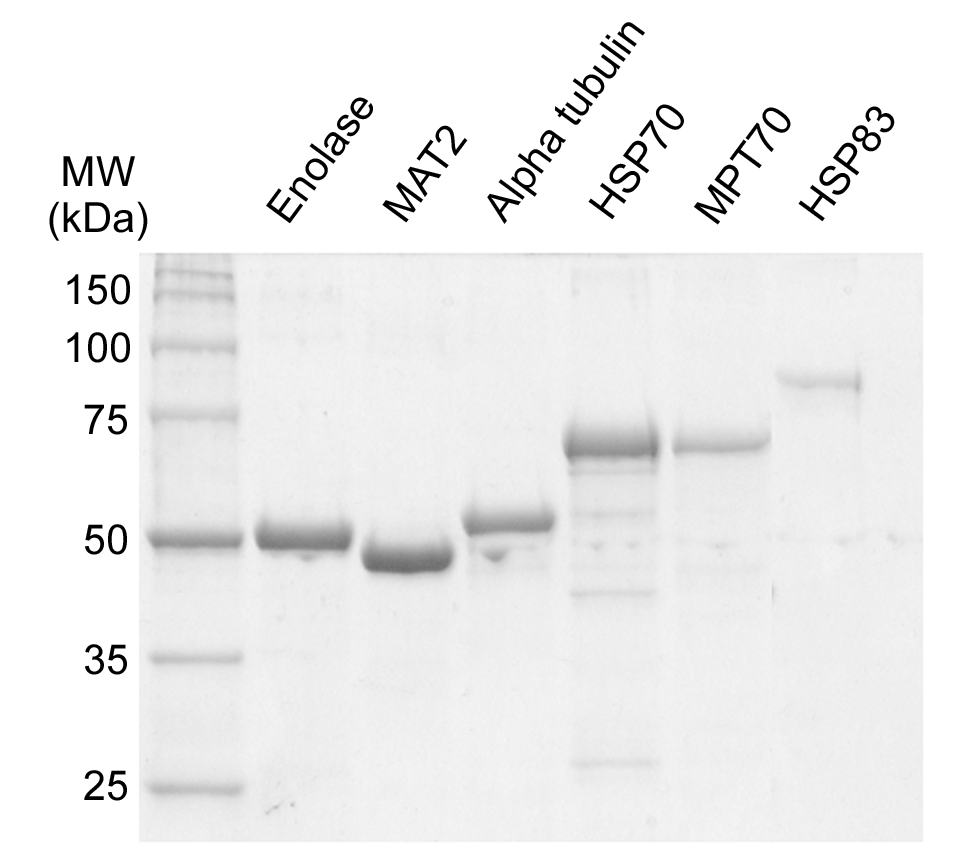

Supplement: Figure S3 [file pntd.0001687.s003.tif]
