# Supplementary material for: Immunodominant Antigens of Leishmania chagasi Associated with Protection against Human Visceral Leishmaniasis
Source: PLoS Negl Trop Dis. 2012 Jun 19;6(6):e1687. doi: 10.1371/journal.pntd.0001687 (PMC3378602; doi:10.1371/journal.pntd.0001687)
Supplement: Table S1 [file pntd.0001687.s004.docx]

**Table S1. Baseline Characteristics of the Study Population.**

| **VL** | | | | | | **Post-VL** | | | | | | | **Asymptomatics** | | | | | |
| --- | --- | --- | --- | --- | --- | --- | --- | --- | --- | --- | --- | --- | --- | --- | --- | --- | --- | --- |
| **No.** | **Age**  **(Yrs)** | **Sex** | **Serum**  **Code** | **DTH** | **ELISA**  **(OD)** | **No.** | **Age**  **(Yrs)** | **Sex** | **Serum**  **Code** | **DTH** | **ELISA**  **(OD)** | **Time post**  **Treatment**  **(Months)** | **No.** | **Age**  **(Yrs)** | **Sex** | **Serum**  **Code** | **DTH** | **ELISA**  **(OD)** |
| 1 | 5 | M | VL732 | - | 0.434 | 1 | 5 | F | 22 | 12.5 | 0.347 | 6 | 1 | 8 | F | 11 | 6.5 | 0.288 |
| 2 | 1 | M | VL731 | - | 0.998 | 2 | 9 | M | 34 | 11.8 | 0.361 | 12 | 2 | 12 | M | 94 | 5.6 | 0.224 |
| 3 | 3 | M | VL678 | - | 1.322 | 3 | 10 | F | 35 | 18.0 | 0.100 | 24 | 3 | 11 | M | 113 | 9.8 | 0.264 |
| 4 | 16 | M | VL671 | - | 1.466 | 4 | 9 | F | 30 | 8.7 | 0.125 | 18 | 4 | 6 | F | 241 | 6.5 | 0.222 |
| 5 | 4 | M | VL666 | - | 0.462 | 5 | 7 | M | 1 | 10.0 | 0.187 | 20 | 5 | 4 | F | 417 | 5.3 | 0.254 |
| 6 | 15 | M | VL660 | - | 1.868 | 6 | 6 | M | 4 | 14.0 | 0.361 | 8 | 6 | 9 | M | 421 | 6.4 | 0.285 |
| 7 | 5 | M | VL667 | - | 0.534 | 7 | 8 | M | 26 | 13.2 | 0.069 | 22 | 7 | 5 | F | 635 | 6.5 | 0.203 |
| 8 | 2 | M | VL669 | - | 0.462 | 8 | 4 | F | 14 | 15.8 | 0.079 | 19 | 8 | 6 | M | 689 | 22.1 | 0.184 |
| 9 | 5 | M | VL663 | - | 0.580 | 9 | 8 | M | 15 | 8.4 | 0.190 | 28 | 9 | 10 | F | 703 | 7.6 | 0.281 |
|  |  |  |  |  |  | 10 | 5 | M | 753 | 11.5 | 0.043 | 6 | 10 | 7 | F | 576 | 9.8 | 0.236 |
|  |  |  |  |  |  | 11 | 1 | M | 728 | 10.0 | 0.174 | 3 | 11 | 3 | M | 618 | 6.5 | 0.237 |
|  |  |  |  |  |  | 12 | 6 | M | 2 | 14.4 | 0.076 | 18 | 12 | 12 | M | 536 | 6.3 | 0.202 |

**Note.** All VL patients presented a positive parasitological test (see Materials and Methods). ELSA´s cut-off: 0.050 using sera diluted 1:100.
